# Bovine *Delta* Papillomavirus E5 Oncoprotein Interacts With TRIM25 and Hampers Antiviral Innate Immune Response Mediated by RIG-I-Like Receptors

**DOI:** 10.3389/fimmu.2021.658762

**Published:** 2021-06-10

**Authors:** Francesca De Falco, Anna Cutarelli, Ivan Gentile, Pellegrino Cerino, Valeria Uleri, Adriana Florinela Catoi, Sante Roperto

**Affiliations:** ^1^ Dipartimento di Medicina Veterinaria e Produzioni Animali, Università degli Studi di Napoli Federico II, Napoli, Italy; ^2^ Istituto Zooprofilattico Sperimentale del Mezzogiorno, Portici, Italy; ^3^ Dipartimento di Medicina Clinica e Chirurgia, Università degli Studi di Napoli Federico II, Napoli, Italy; ^4^ Physiopathology Department, Faculty of Medicine “Iuliu Hatieganu”, University of Medicine and Pharmacy, Cluj-Napoca, Romania

**Keywords:** bovine papilloma virus E5 oncoprotein, tripartite motif containing 25 (TRIM25), retinoic acid-inducible gene I (RIG-I), mitochondrial antiviral-signalling (MAVS) protein, melanoma differentiation-associated gene 5 (MDA5)

## Abstract

Persistent infection and tumourigenesis by papillomaviruses (PVs) require viral manipulation of various of cellular processes, including those involved in innate immune responses. Herein, we showed that bovine PV (BPV) E5 oncoprotein interacts with a tripartite motif-containing 25 (TRIM25) but not with Riplet in spontaneous BPV infection of urothelial cells of cattle. Statistically significant reduced protein levels of TRIM25, retinoic acid-inducible gene I (RIG-I), and melanoma differentiation-associated gene 5 (MDA5) were detected by Western blot analysis. Real-time quantitative PCR revealed marked transcriptional downregulation of RIG-I and MDA5 in E5-expressing cells compared with healthy urothelial cells. Mitochondrial antiviral signalling (MAVS) protein expression did not vary significantly between diseased and healthy cells. Co-immunoprecipitation studies showed that MAVS interacted with a protein network composed of Sec13, which is a positive regulator of MAVS-mediated RLR antiviral signalling, phosphorylated TANK binding kinase 1 (TBK1), and phosphorylated interferon regulatory factor 3 (IRF3). Immunoblotting revealed significantly low expression levels of Sec13 in BPV-infected cells. Low levels of Sec13 resulted in a weaker host antiviral immune response, as it attenuates MAVS-mediated IRF3 activation. Furthermore, western blot analysis revealed significantly reduced expression levels of pTBK1, which plays an essential role in the activation and phosphorylation of IRF3, a prerequisite for the latter to enter the nucleus to activate type 1 IFN genes. Our results suggested that the innate immune signalling pathway mediated by RIG-I-like receptors (RLRs) was impaired in cells infected with BPVs. Therefore, an effective immune response is not elicited against these viruses, which facilitates persistent viral infection.

## Introduction

Pattern recognition receptors (PRRs) are responsible for sensing the presence of pathogens, including viruses, since they recognise conserved features of microbes known as pathogen-associated molecular patterns (PAMPs) (1). Four different classes of PRRs have been identified: Toll-like receptors (TLRs), C-type lectin receptors (CLRs), retinoic acid-inducible gene I (RIG-I)-like receptors (RLRs), and NOD-like receptors (NLRs) (2).

RLRs are PRRs expressed both in professional and in various non-professional immune cells, including epithelial cells (2). RLRs play a major role in triggering and modulating antiviral immunity by detecting exogenous viral RNAs (3, 4). The RLR family is composed of retinoic acid-inducible gene I (RIG-I), melanoma differentiation-associated gene 5 (MDA5), and laboratory of genetics and physiology 2 (LGP2). RLRs localise to the cytosol, and their expression is maintained at low levels in resting cells but is greatly increased after virus infection (3, 5). The activation of RIG-I and MDA5 is regulated by multiple ubiquitin E3 ligases of the tripartite motif (TRIM) proteins such as TRIM containing 25 (TRIM25) and Riplet (6). RIG-I and MDA5 sense viral RNAs through the mitochondrial antiviral signalling (MAVS) protein (7). Although the majority of MAVS is present on the outer mitochondrial membrane (OMM), a small proportion is located in the mitochondria-associated membranes (MAMs) as well as in the peroxisomes (8). RIG-I and MDA5 harbour caspase activation and recruitment domains (CARDs), and they bind to and activate MAVS *via* CARD-CARD interactions, triggering polymerisation of MAVS into prion-like structures required for antiviral signalling (9, 10). Activation of MAVS on mitochondria and MAMs results in stimulation of the kinases TBK1 and IKK and, consequently, of the transcription factors IRF3, IRF7, and NF-κB for the induction of genes encoding type I and type III interferon and pro-inflammatory cytokines (5, 11, 12). LGP2 lacks antiviral signalling activity. LGP2 has been proposed to be an accessory protein important for regulating RIG-I and MDA5 signalling (5). Indeed, LGP2 interacts with MAVS in microsomes, blocking RIG-I/MAVS. After virus infection, LGP2 is rapidly released from MAVS and redistributed to mitochondria, which correlates with IRF3 activation (13).

Besides RNA ligands from RNA viruses, RLRs recognise DNA ligands from DNA viruses as well as those derived from bacteria (5). RLRs are known to detect herpesviruses, adenoviruses, and poxviruses (5, 8, 14). Recently, it has been shown that high-risk human papillomaviruses (HPVs) can downregulate RLR expression, thus creating a cellular milieu suitable for their persistence (15).

Bovine papillomaviruses (BPVs), a heterogeneous group of species-specific viruses distributed worldwide (16), comprise 29 types assigned to five genera (17). Bovine δPVs are known to infect epithelial and mesenchymal cells and are unique among BPVs to show cross-species transmission and infection (16). Besides skin tumours, bovine δPVs play a very important role in the bladder carcinogenesis of large ruminants, such as cattle and buffaloes (18, 19). Bovine δPVs show their transforming activity through the E5 protein, a highly conserved oncoprotein, believed to be the major δPV oncoprotein (20). E5 forms dimers and displays pathogen activity *via* numerous pathways in the absence of other viral genes (20). E5 can bind to the activated form of platelet-derived growth factor ß receptor (PDGFßR) (19, 21). E5 can also act *via* the calpain3 pathway and/or it binds to the subunit D of the V_1_-ATPase proton pump (22, 23). E6 and E7 are less studied δPV oncoproteins.

We aimed to investigate the interaction between E5 oncoprotein and E3 ubiquitin ligase TRIM25 and the downregulation of multiple downstream effectors of the host antiviral response pathway mediated by the RLRs in a spontaneous model of bovine papillomavirus disease.

## Materials and Methods

### Animal Samples

Bladder mucosa samples from 15 cows clinically suffering from chronic enzootic haematuria were collected from public slaughterhouses after bladder neoplasms had been identified during mandatory post-mortem examination. These animals were categorised as “infected”. Bladder mucosa samples from 15 apparently healthy cows were also collected. Six of these apparently healthy bladders showed inflammatory cells composed of small foci of lymphocytes beneath the urothelium. These animals were categorised as “Non-infected” as they did not harbour any papillomavirus infection. The remaining nine animals were categorised as “healthy” since no inflammatory cells were seen in their bladder samples. Six of these apparently heathy bladders showed small foci of lymphocytes beneath the urothelium. Animals from both groups were 3-5 years old. All bladder samples were immediately subdivided and either fixed in 10% buffered formalin for microscopic investigation or frozen in liquid nitrogen and stored at –80°C for subsequent molecular biology analysis.

### Antibodies

Rabbit antibodies against RIG-1, MDA5, IRF3, phospho-IRF3, TBK1, phospho-TBK1, and TRIM25, were obtained from Cell Signaling Technology (Leiden, Netherlands). Rabbit antibody anti-RNF135 (RIPLET) was purchased from Sigma-Aldrich (MO, USA). Mouse anti-MAVS, anti-Sec13, and b-actin antibodies were purchased from Santa Cruz Biotechnology (TX, USA). Rabbit polyclonal anti-E5 serum recognising the C-terminal 14 amino acids of the BPV E5 oncoprotein was kindly gifted provided by Prof. DiMaio (Yale University, New Haven USA).

### RNA Extraction and Reverse Transcription (RT)-PCR

Total RNA was extracted from bladder samples from 15 cows suffering from chronic enzootic haematuria and 10 healthy cows using the RNeasy Mini Kit (Qiagen, NW, DE), according to the manufacturer’s instructions. Genomic DNA was removed from the RNA preparations using RNase-free DNase Fermentas Life Sciences (Thermo Fisher Scientific, MA, USA). A total of 1 μg RNA was used to generate single-stranded cDNA, using the QuantiTect Reverse Transcription Kit (Qiagen NW, DE), according to the manufacturer’s instructions. PCR was performed with a specific primer set designed using Primer3, an online tool, for BPV-2 E5, BPV-13 E5, bovine RIG-I, MDA5, and TRIM25 genes. The following primers were used: *BPV-2 E5* ORF forward 5′-CACTGCCATTTGTTTTTTTC-3′, reverse 5′-GGAGCACTCAAAATGATCCC-3′; *BPV-13 E5* ORF forward 5′-CACTGCCATTTGGTGTTCTT-3′, reverse 5′- AGCAGTCAAAATGATCCCAA-3′; *RIG-I* forward 5’- AGGAAAAGATTCGCCAGATACAGA-3’; reverse 5’-ATGGCATTCCTCCACCACTC-3’; *MDA5* forward 5’-TGAAGCAGGGGTAAGAGAGC-3’; reverse 5’-TCAGACTCTGTACTGCCTTCAC-3’: *TRIM25* forward 5’- CGGAGCTCCTGGAGTATGTG-3’; reverse 5’- TAGTTCAGGGATGCGTCAGC -3’. *IKKα* forward: 5’-CTCAGAGTTCTGCTCGGTCC-3’; reverse: 5’-AGTCTCCCCATCTTGAGGAGTT-3’; *IKKβ*; forward 5’- CAGAAGAGCGAGATGGACATTG-3’; reverse 5’-CCAGGACGCTGTTGAGGTT-3’: *IKKγ:* forward 5’-GTGAGCGGAACCGAGGAC-3’; reverse: 5’- CTGGGCTTTTAGCACTGGGA-3’. *IFN-β:* forward 5’- TCGGCATTCTCACCAGAGAC-3’; reverse: 5’-GGAACGATCGTGTCTTCCGT-3’. Conditions for PCR were as follows: 94°C for 5 min, 40 cycles at 95°C for 30 s, 58°C for 30 s, and 72°C for 30 s.

### One-Step Reverse Transcription (RT)-ddPCR

Total RNA was extracted from 15 bovine urothelial tumour samples and 3 bladder samples from healthy cows (as negative control) as previously reported. 100ng of total RNA was used for One-Step RT-ddPCR Advanced Kit for Probes (Bio-Rad), according to the manufacturer’s instructions. The reaction was performed in a final volume of 20 μL containing: 10 μL of ddPCR Supermix for Probes, 0.9 μM primer for BPV2-E5, 0.25 μM probe, 2 ml Reverse transcriptase, 300nM DTT. The following primers were used: BPV-2 E5 Forward: 5’TACAGGTCTGCCCTTTTAAT 3’; Reverse: 5’AACAGTAAACAAATCAAATCCA3’; probe: 5’AACAACAAAGCCAGTAACC 3’ FAM.

A black hole quencher was used in combination with FAM fluorescent dye reporters (Bio-Rad Laboratories). The reaction mixture was placed into the sample well of a DG8 cartridge (Bio-Rad Laboratories). A volume of 70 μL of droplet generation oil was loaded into the oil well, and droplets were formed in the droplet generator (Bio-Rad Laboratories). After processing, the droplets were transferred to a 96-well PCR plate (Eppendorf, Hamburg, Germany). PCR amplification was carried out on a T100 Thermal Cycler (Bio-Rad Laboratories) with the following thermal profile: 50°C for 60min, 95 °C for 10 min, 40 cycles of 94°C for 30 s and 56°C for 1 min, 1 cycle at 98°C for 10 min, and ending at 4°C. After amplification, the plate was loaded onto a droplet reader (Bio-Rad Laboratories) and the droplets from each well of the plate were read automatically. QuantaSoft software was used to count the PCR-positive and PCR-negative droplets to provide absolute quantification of the target cDNA. Therefore, the ddPCR results could be directly converted into copies/µL in the initial samples simply by multiplying them by the total volume of the reaction mixture (20 µL) and then dividing that number by the volume of RNA sample added to the reaction mixture (5 µL) at the beginning of the assay. Each sample was analysed in quadruplicate.

### Real-Time RT- PCR

To perform real-time RT-PCR analysis, total RNA and cDNA from diseased and healthy urinary bladder samples were generated, as described above. Real-time PCR was performed with a Bio-Rad CFX Connect™ Real-Time PCR Detection System (Bio-Rad, Hercules, CA, USA), using iTAq Universal SYBR^®^ Green Supermix (Bio-Rad). Each reaction was performed in triplicate, and the primers used for RIG-I, MDA5, TRIM 25, IKKα, IKKβ, IKKγ, and IFN-β were the same as those used for RT-PCR. The PCR thermal profile was as follows: 95°C for 10 min, 40 cycles of 94°C for 15 s, and 58°C for 30 s, followed by a melting curve. Relative quantification (RQ) was calculated using the CFX Manager™ software, based on the equation RQ=2−ΔΔCq, where Cq is the quantification cycle to detect fluorescence. Cq data were normalised to the bovine *β-actin* gene (forward: 5′- TAGCACAGGCCTCTCGCCTTCGT-3′, reverse 5′-GCACATGCCGGAGCCGTTGT-3′).

### Sequence Analysis

PCR products, obtained by RT-PCR, were purified using the QIAquick PCR Purification Kit 131 (Qiagen NW, DE) and were subjected to bidirectional sequencing using the Big Dye-Terminator v1.1 Cycle 132 Sequencing Kit (Applied Biosystems, CA, USA), according to the manufacturer’s recommendations. Dye terminators from 133 sequences were removed using a DyeEx-2.0 Spin Kit (Qiagen), and sequences were run on a SeqStudio 134 Genetic Analyzer (Thermo Fischer Scientific, CA, USA). Electropherograms were analysed using Sequencing Analysis v5.2 and Sequence Scanner v1.0 softwares (Thermo Fischer Scientific, CA, USA). The sequences were analysed using the BLAST program.

### Western Blot Analysis

Healthy and diseased bovine urothelial samples were lysed in radioimmunoprecipitation assay (RIPA) buffer (50 mM Tris-HCl [pH 7.5], 1% Triton X-100, 400 mM NaCl, 1 mM 151 ethylenediaminetetraacetic acid, 2 mM phenylmethylsulfonyl fluoride, 1.7 mg/mL aprotinin, 50 mM 152 NaF, and 1 mM sodium orthovanadate). Protein concentration was measured using the Bradford assay (Bio-Rad). For western blotting, 50 μg protein lysate was heated at 90°C in 4X premixed Laemmli sample buffer (Bio-Rad), clarified by centrifugation, separated by sodium dodecyl sulphate–polyacrylamide gel electrophoresis, and transferred onto nitrocellulose membranes (GE Healthcare, UK). Membranes were blocked with Tris-buffered saline and 0.1% Tween 20 (TBST)- containing 5% bovine serum albumin (BSA) for 1 h at room temperature. The membranes were subsequently incubated overnight at 4°C with primary antibodies, washed three times with TBST, incubated for 1 h at room temperature with goat anti-rabbit or goat anti-mouse (Bio-Rad) HRP- conjugated secondary antibody, diluted at 1:5,000 in TBST containing 5% BSA, and washed three times with TBST. Immunoreactive bands were detected using Western Blotting Luminol Reagent (Santa Cruz Biotechnology) and ChemiDoc XRS Plus (Bio-Rad). Images were acquired using Image Lab Software version 2.0.1.

### Immunoprecipitation

Total protein extracts from normal and pathological bladders, obtained as previously described, were immunoprecipitated. Protein samples (1mg) were incubated with anti-TRIM25 or anti-rabbit IgG (isotype), anti-Riplet or anti-rabbit IgG (isotype), and anti-MAVS or anti-mouse IgG antibodies (Bethyl Laboratories, Inc., TX, USA) for 1 h at 4°C with gentle shaking. Thereafter, the samples were centrifuged at 1,000 *g* for 5 min at 4°C and incubated with 30 μL of Protein A/G-Plus Agarose (sc-184 2003) (Santa Cruz Biotechnology) overnight at 4°C. The immunoprecipitates were washed four times in complete lysis buffer and separated on polyacrylamide gels. Subsequently, the proteins were transferred onto nitrocellulose membranes. The membranes were blocked for 1 h at room temperature (25°C) in TBST with 5% BSA, and then incubated with primary antibodies overnight at 4°C. After three washes in TBST, the membranes were incubated with secondary antibodies for 1 h at room temperature. Chemiluminescent signals were developed using the Western Blotting Luminol Reagent (Santa Cruz Biotechnology) and were detected using the ChemiDoc XRS gel documentation system (Bio-Rad).

### Statistical Analysis

Results are presented as the mean ± standard error (SE). Data were assessed by one-way analysis of variance (ANOVA), followed by Tukey’s test for multiple comparisons of means using the GraphPad PRISM software version 9 (GraphPad Software, San Diego, CA, USA). A p-value ≤ 0.05 indicated statistical significance. To analyse the relationship between protein or mRNA expression and BPV-2 E5 viral load, Spearman’s and Pearson’s correlation analyses were performed for protein or mRNA expression and BPV-2 E5 cDNA load in the same samples using GraphPad PRISM software version 9 (GraphPad Software, San Diego, CA, USA). A p-value ≤ 0.05 indicated statistical significance.

## Results

### Virological Findings

**Figure 1:**
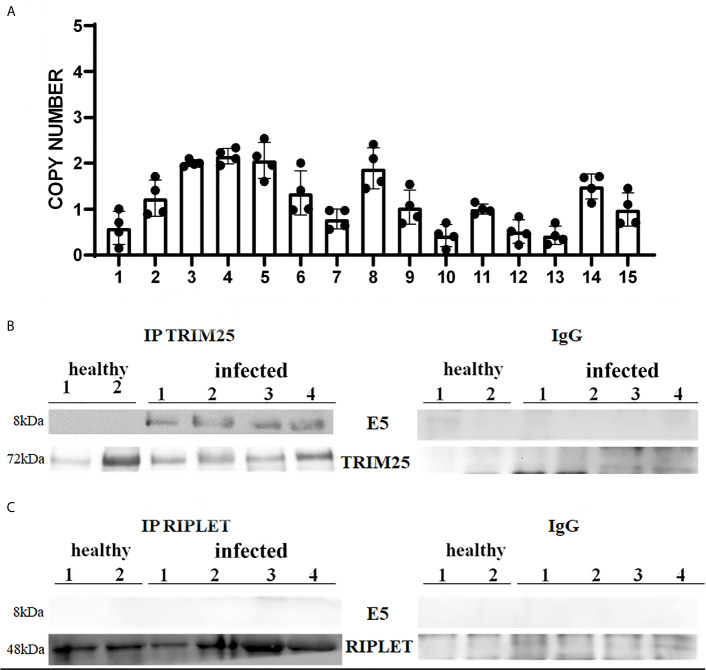
**(A)** Copy number of BPV E5 mRNA by one-step RT-ddPCR analysis detected in 15 bladder tumours. Each plot represents an independent experiment. Statistical analysis revealed no significant differences in copy number of BPV E5 mRNA of each analysed bladder tumours. **(B, C)** showed immunoprecipitation assay using anti-TRIM25 and anti-Riplet antibodies, respectively, in healthy and infected bladder samples. Western blot analysis revealed that TRIM25 only interacted with E5 protein. Panels **(B, C)** show representative data from three independent experiments.

It is well known that bovine δPVs are the most important infectious agents involved in the etiopathogenesis of the majority of bovine urothelial tumours (18, 24). E5 oncoprotein expression is correlated with the transformation of both mesenchymal and epithelial cells to form benign and malignant tumours (25). Therefore, we attempted to verify whether the δPV E5 oncoprotein was expressed in the examined samples. First, we detected E5 oncoprotein transcripts by RT-PCR, the sequencing of which showed 100% identity with BPV-2 and BPV-13 sequences deposited in GenBank (Accession numbers M20219.1 and JQ798171.1, respectively) (**Supplemental Figure 1**). Furthermore, Western blot analysis revealed the expression of E5 oncoprotein, which showed that abortive infection takes place (data not shown). We used One-Step reverse transcription (RT)-ddPCR tool to investigate the copy number of E5 oncoprotein mRNA in 15 cattle suffering from bladder cancer caused by persistent BPV infections (26). DdPCR method was able to detect and quantify E5 mRNA in cancer urothelial cells from all 15 cattle, however, no statistically significant differences in copy number of E5 mRNA were found (**Figure 1A**).

### Expression of Tripartite Motif-Containing 25 (TRIM25) and Riplet Proteins

As many viruses, including human papillomavirus, have E3 ubiquitin ligases as their targets (27), we wondered whether the bovine δPV E5 oncoprotein might interact with some ligases involved in the antiviral innate immune response mediated by RLRs, the ubiquitination of which appears to be a key post-translational modification. However, the molecular mechanisms of ubiquitin-mediated RIG-I and MDA5 activation remain to be fully understood (28, 29).

Many studies have reported that TRIM25 and Riplet are two essential E3 ubiquitin ligases for RIG-I signalling as they are known to ubiquitinate and activate RLRs (6, 29).

**Figure 2:**
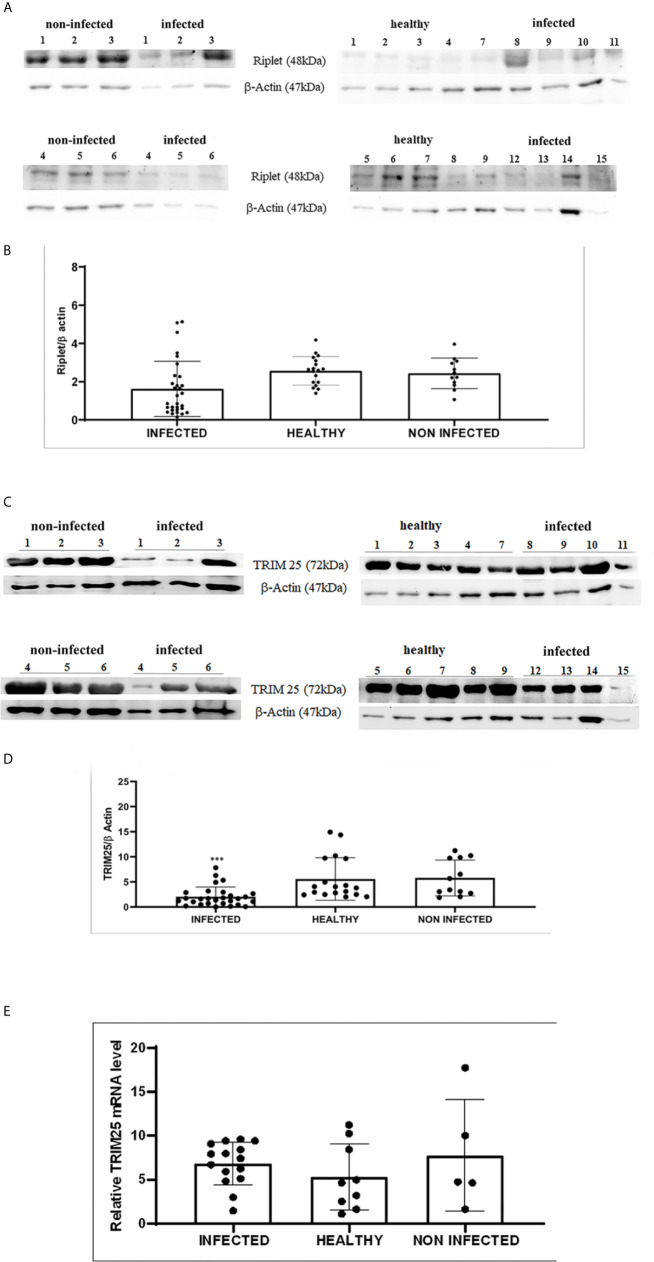
**(A)** Western blot analysis of total Riplet protein in infected, healthy and non-infected but inflammatory bladder samples. **(B)** Densitometric analysis of total Riplet protein relative to the β-actin protein level. Plots represent value found in each sample performed in duplicate. **(C)** Western blot analysis of total TRIM25 protein in infected, healthy and non-infected but inflammatory bladder samples. **(D)** Densitometric analysis of total TRIM25 protein relative to the β-actin protein level (*** p ≤ 0.001). Plots represent value found in each sample performed in duplicate. **(E)** Real time RT-PCR. TRIM25 mRNA levels in infected, healthy and non-infected but inflammatory bladder samples. Plots represent value found in each sample performed in triplicate. Statistical analysis revealed no significant difference between value of each pathological bovine bladder compared to the value of both healthy and uninfected bladders.

Therefore, we investigated these two ligases by performing co-immunoprecipitation studies using anti-TRIM25 and anti-Riplet antibodies. The assay revealed the presence of E5 oncoprotein in anti-TRIM25 immunoprecipitates only, suggesting that the E5 oncoprotein of bovine δPVs interacts with TRIM25 but not with Riplet (**Figures 1B, C**). Our results are in line with *in vitro* studies performed on cells experimentally infected with HPV18, which showed that TRIM25 but not Riplet was a target of viral E6 oncoprotein (30). We then investigated the expression levels of these two ligases in 15 bladder samples from cattle cancer. Furthermore, we studied these two ligases in additional 15 bladder samples from uninfected, apparently healthy cattle, six of which showed small foci of lymphocytes beneath the normal urothelium. Western blot analysis of total extracts detected unmodified levels of Riplet expression (**Figures 2A, B**) and a statistically significant reduction in the expression of TRIM25 (**Figures 2C, D**) in neoplastic bladder cells in comparison with all apparently healthy bladder cells. To understand whether the marked reduction in TRIM25 expression levels could be attributed to transcriptional events and/or increased protein degradation, we investigated the presence of TRIM25 transcripts by RT-PCR. Sequencing of the obtained cDNA amplicons showed 100% identity with bovine TRIM25 sequences deposited in GenBank (Accession number: NM_001100336.1) (**Supplemental Figure 2**). Then, we performed a real-time PCR analysis on cDNA using specific primers for bovine TRIM25. This molecular assay did not show any variation in transcript expression in cells infected with bovine δPVs compared with cells from clinically normal cattle (**Figure 2E**). These results suggest that bovine δPVs interfere at the protein level rather than at the transcriptional level in reducing TRIM25 expression.

### Expression Levels of RIG-I and MDA5 and Their Downstream Effectors

Expression of RLRs is ubiquitous and is typically maintained at low levels in resting cells but is greatly increased after virus infection (3). Therefore, we decided to investigate RLR expression during spontaneous BPV infection.

**Figure 3:**
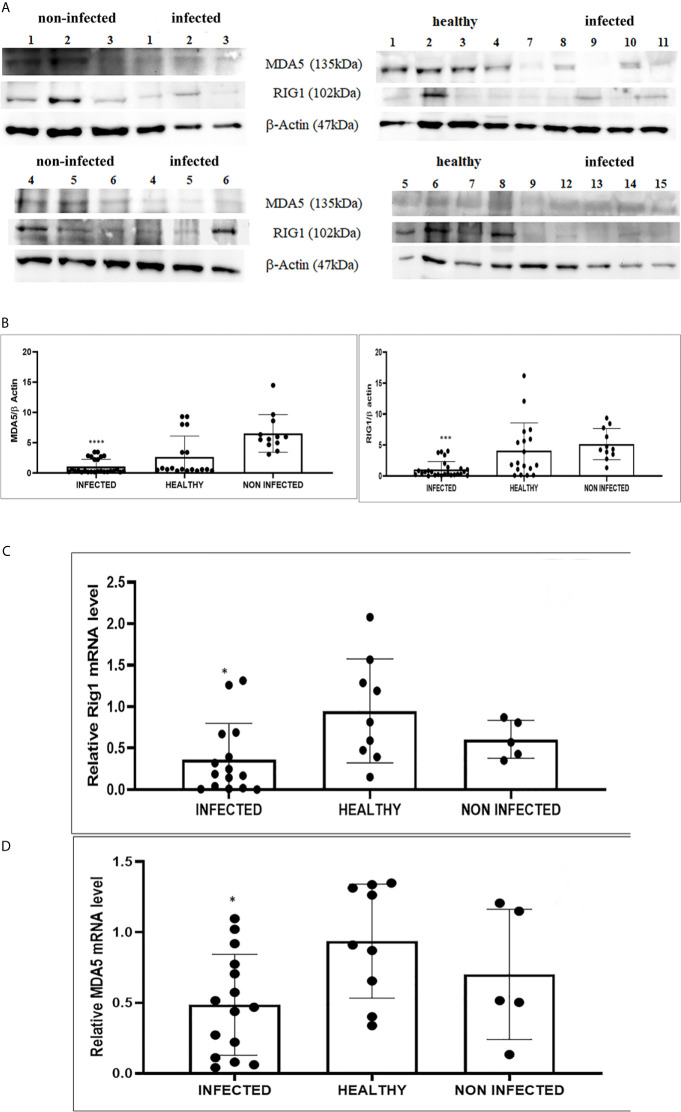
**(A)** Western blot analysis of RIG-1 and MDA5 in infected, healthy and non-infected but inflammatory bladder samples. **(B)** Densitometric analysis was performed by comparing the protein expression levels of total RIG-1 and MDA5 with those of β-actin. RIG-1 and MDA5 protein levels were significantly reduced in the infected mucosa samples compared with both healthy and uninfected samples. (***p ≤ 0.001; ****p ≤ 0.0001). Plots represent value found in each sample performed in duplicate. **(C, D)** Real-time RT-PCR analysis of RIG-I and MDA5 mRNA levels, respectively, in infected, healthy and non-infected but inflammatory bladder samples. RIG-I and MDA5 mRNA expressions were significantly reduced in diseased bladder samples compared with both healthy and uninfected bladder (*p ≤ 0.05). Plots represent value found in each sample performed in triplicate.

We detected reduced expression levels of both RIG-I and MDA5 by Western blot analysis in urothelial cells infected by bovine δPVs compared with urothelial cells from clinically normal cattle including six animals showing foci of lymphocytes beneath the normal urothelium (**Figures 3A, B**). We assumed that the levels of these proteins could be due to transcriptional reduction. Using specific primers for bovine RIG-I and MDA5, we carried out a real-time PCR. Sequencing of the transcript amplicons revealed cDNA fragments showing 100% identity with bovine RIG-I and MDA5 sequences deposited in GenBank (Accession numbers: XM_002689480.6 and XM_010802053.2, respectively) (**Supplemental Figure 3**). Real-time PCR of cDNA revealed a statistically significant reduction in both RIG-I and MDA5 transcripts in δPV-positive cells compared with all δPV-negative cells (**Figures 3C, D**). These results suggest that, like HPVs, bovine δPVs may interfere at the transcriptional level rather than at the protein level in reducing RIG-I and MDA5 expression to prevent their antiviral activities.

**Figure 4:**
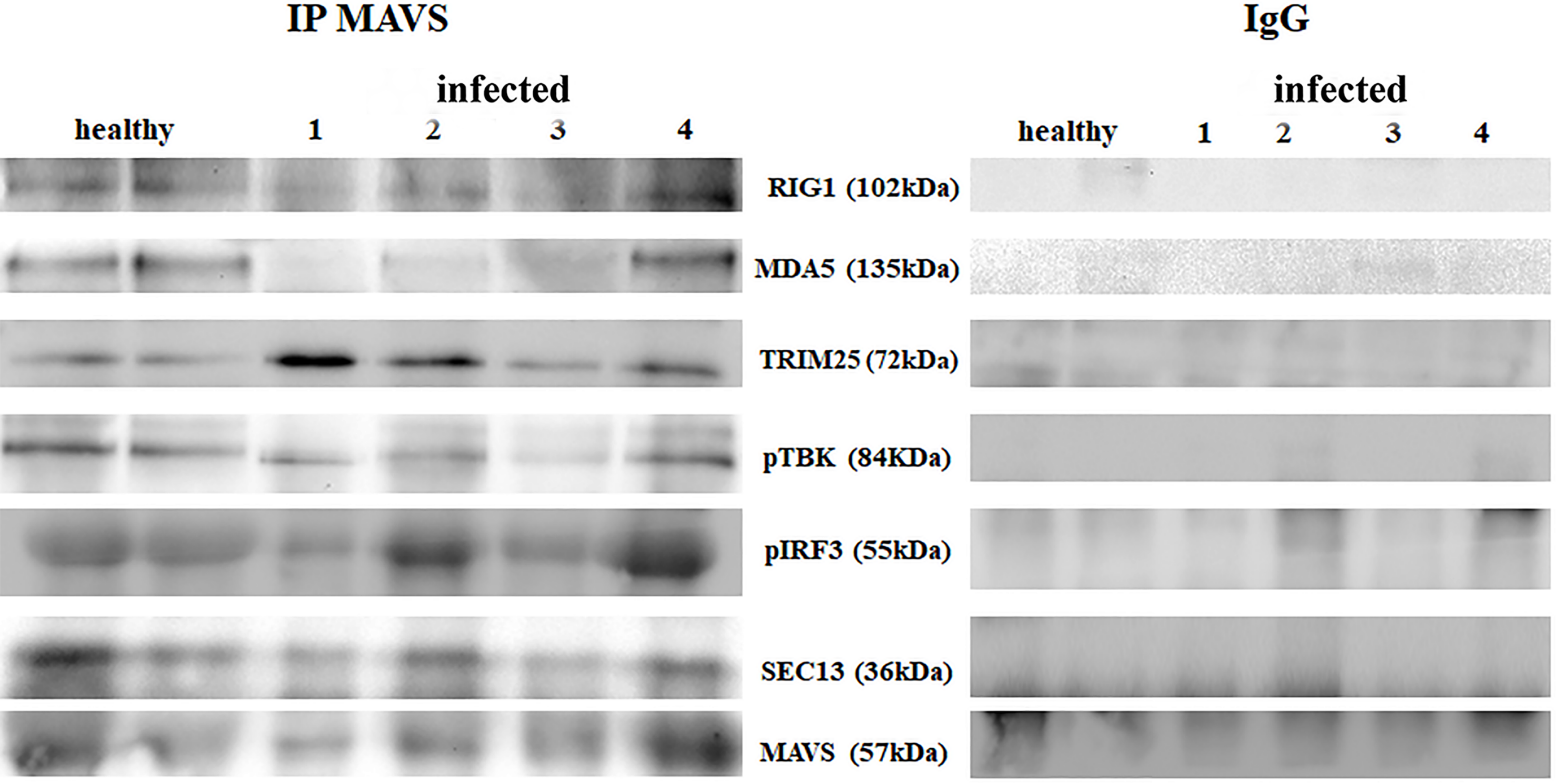
Immunoprecipitation using an anti-MAVS antibody in healthy and diseased bladder samples. Western blot analysis revealed that MAVS interacted with RIG-I, MDA5, TRIM25, phosphorylated TBK1 (pTBK1), phosphorylated IRF3 (pIRF3) and Sec13. Immunoprecipitation panel showed representative data from three independent experiments.

**Figure 5:**
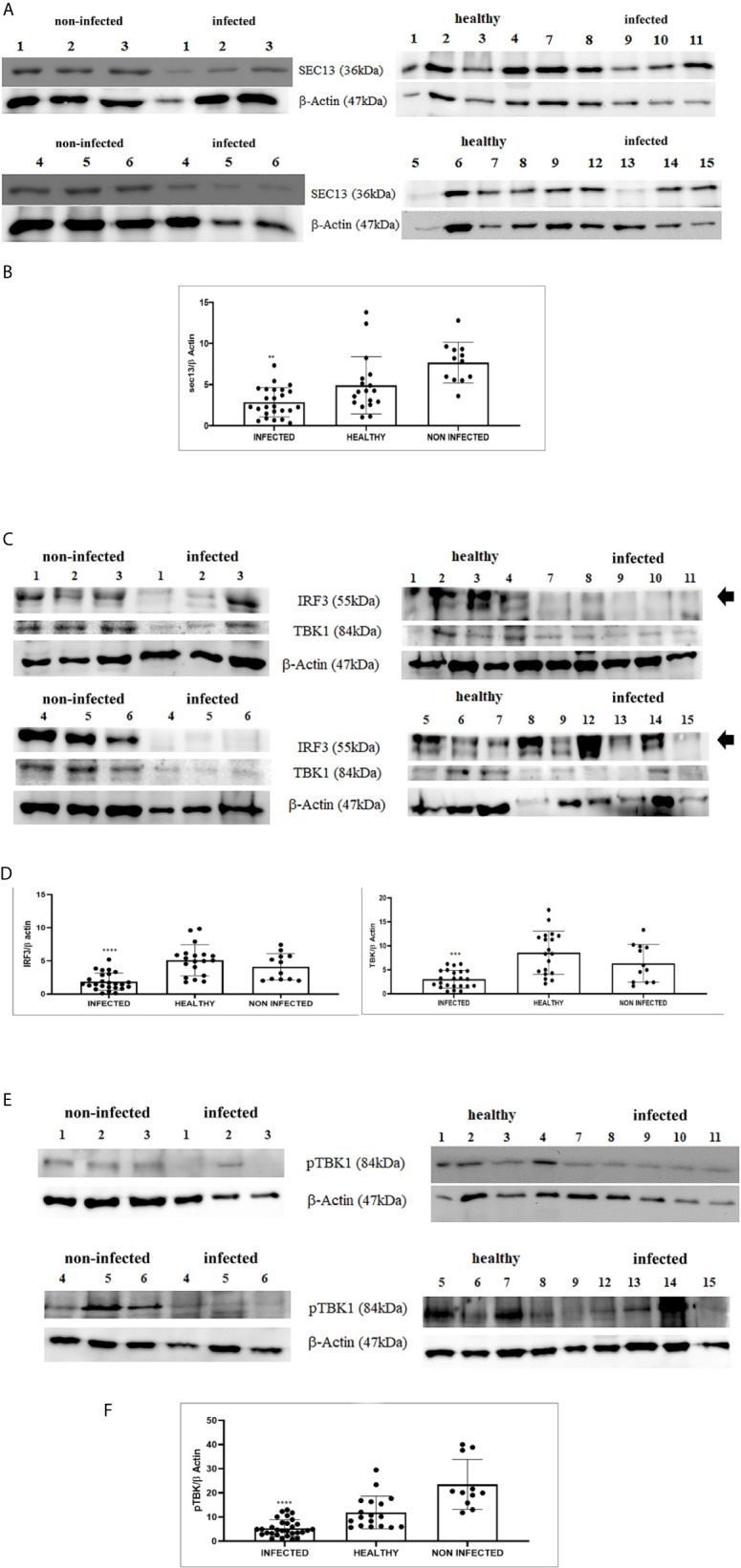
**(A)** Western blot analysis of total Sec13 protein in infected, healthy and non-infected but inflammatory bladder samples. **(B)** Densitometric analysis of total Sec13 protein relative to the β-actin protein level (**p ≤ 0.01). Plots represent value found in each sample performed in duplicate. **(C)** Western blot analysis of IRF3 and TBK1 proteins in infected, healthy and non-infected but inflammatory bladder samples. **(D)** Densitometric analysis of both proteins was performed relative with the β-actin protein levels (***p ≤ 0.001; ****p ≤ 0.0001). IRF3 band used for densitometry was indicated by arrow. Plots represent value found in each sample performed in duplicate. **(E)** Western blot analysis of phosphorylated TBK1 (pTBK1) in infected, healthy and non-infected but inflammatory bladder samples. **(F)** Densitometric analysis of pTBK1 protein relative to β-actin protein levels (****p ≤ 0.0001). Plots represent value found in each sample performed in duplicate.

**Figure 6:**
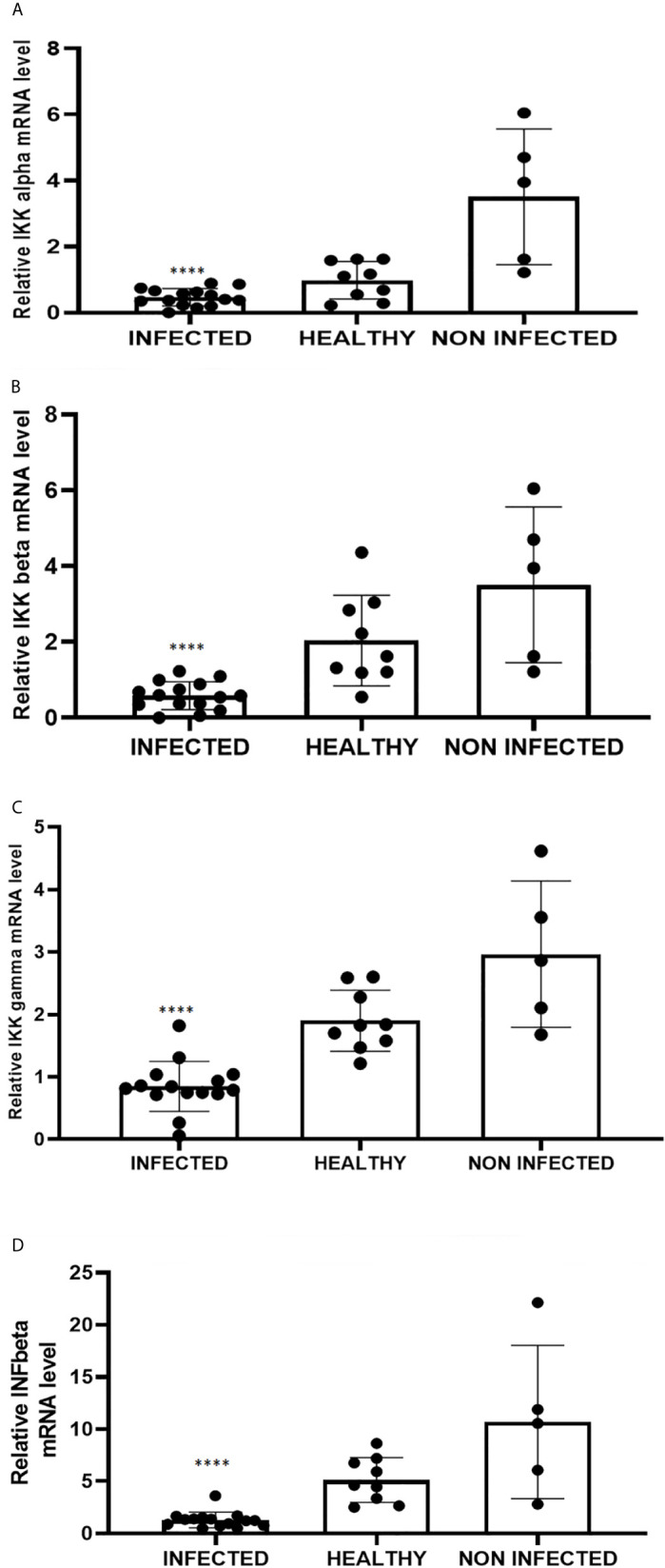
Real-time RT-PCR analysis of **(A)** IKKα, **(B)** IKKβ, **(C)** IKKγ, **(D)** IFN-β mRNA levels in infected, healthy and non-infected but inflammatory bladder samples. IKKα, IKKβ, IKKγ and IFN-β mRNA levels were significantly reduced in diseased bladder samples compared with both healthy and uninfected bladder samples (****p ≤ 0.0001). Plots represent value found in each sample performed in triplicate.

RIG-I and MDA5 interact with a mitochondrial adaptor, the mitochondrial antiviral signalling (MAVS) protein (4, 29). It remains unclear how MAVS acts as a scaffold to assemble the signalosome in RLR-mediated antiviral signalling (31). Western blot analysis of MAVS expression revealed unmodified protein expression levels in both δPV-infected and healthy cells (data not shown). Our results are in line with experimental data showing that the expression levels of MAVS did not significantly vary in cells in which the E6 oncoprotein of HPV18 was shown to act as a RIG-I transcriptional repressor (15). We then performed co-immunoprecipitation studies using an anti-MAVS antibody. Western blot analysis performed on the immunoprecipitates detected the presence of RIG-I and MDA5 as well as TRIM25, phosphorylated TANK-binding protease 1 (pTBK1), phosphorylated interferon regulatory factor 3 (IRF3), and Sec13, which is believed to be a positive regulator of MAVS (31) (**Figure 4**). Western blot analysis performed on total extracts revealed a statistically significant reduction in the expression levels of Sec13 in δPV-infected cells compared with cells from clinically normal cattle (**Figures 5A, B**), which suggests that MAVS activation might be compromised in cells spontaneously infected with bovine δPVs. MAVS subsequently phosphorylates and activates TBK1 and IRF3, *via* an unknown mechanism, which results in the production of interferons as well as proinflammatory factors (32). Western blot analysis performed on anti-MAVS immunoprecipitates revealed the presence of pTBK1 and pIRF3, which suggests that MAVS forms a complex with pTBK1 and pIRF3 and plays a critical role in driving and coordinating synergistic functional activities of these downstream components. Moreover, we investigated the expression levels of TBK1 and IRF3 in total extracts of samples comprising three groups: 9 healthy bladders (BPV-negative, pTBK-low expression levels), 6 uninfected bladders containing small foci of lymphocytes in their wall (BPV-negative, pTBK-high expression levels) and 15 pathological BPV-infected bladders (BPV-positive, independently of pTBK levels). Immunoblotting revealed statistically significant reduced levels of both proteins in cells infected with bovine δPVs compared with uninfected cells (**Figures 5C, D**). Furthermore, western blot analysis revealed statistically significant reduced expression levels of pTBK1 (**Figures 5E, F**). TBK1 is activated *via* phosphorylation (33), which in turn phosphorylates and activates IRF3. Subsequently, IRF3 enters the nucleus to activate type 1 IFN (32, 34). Antiviral cytokines are also under control of nuclear factor kappa B (NF-kB), a family of transcription factors. It has been suggested that NF-kB is rendered inactive because virus infection results in reduced levels of inhibitor of kappa B kinase (IKK) complex, which is needed for NF-kB function (35). The canonical complex of IKK is composed of IKKα/IKKβ/IKKγ. Real time RT-PCR of mRNA of these IKKs showed significant reduced levels of mRNA of all three kinases in urothelial cells infected by BPVs compared to uninfected, heathy urothelial cells (**Figures 6A–C**). Then, we investigated the expression of downstream antiviral cytokines. In particular, we performed real time RT-PCR of mRNA of IFN-β, a cytokine essential for host antiviral responses. We detected a statistically significant reduced levels of INF-β mRNA by real time RT-PCR in urothelial cells infected by BPVs in comparison to uninfected urothelial cells of healthy cattle (**Figure 6D**).

Altogether, our results suggest that the transcriptional downregulation of RIG-I and MDA5 in cells infected with bovine δPVs is responsible for an aberrant downstream signalling pathway, including TBK1/IRF3, and IKKs which may lead to the impairment of the host antiviral response. Then, we asked whether there was a correlation between viral load and expression levels of investigated proteins. Our results showed a lack of a direct correlation (**Supplemental Figures 4** and **5**).

**Figure 7:**
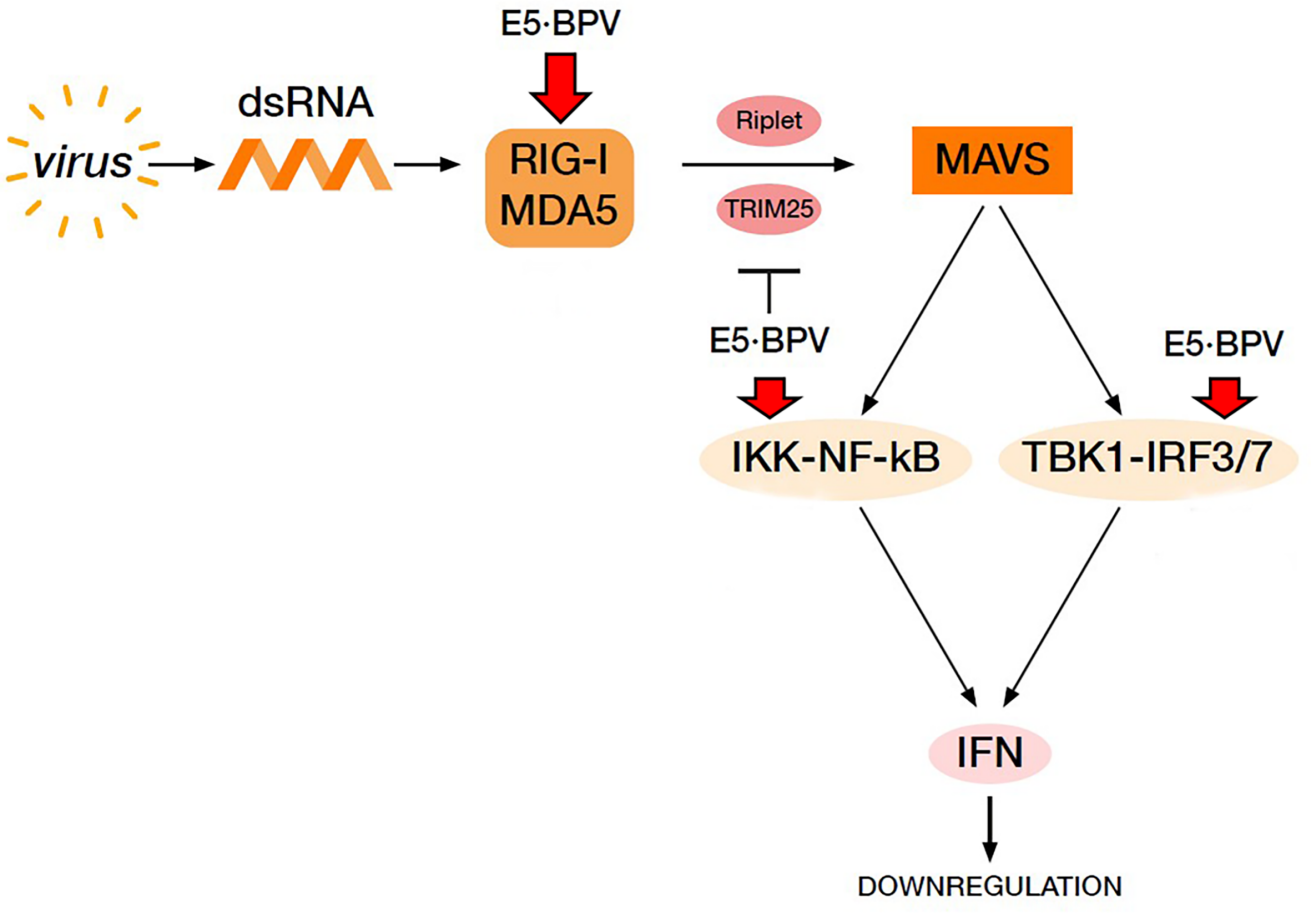
Virus-mediated RIG-I and MDA5 signalling pathway resulting in IFN production. BPV E5 oncoprotein perturbs this pathway leading to a downregulation of IFN production thus contributing to cause a BPV persistent infection. Red arrows indicate downregulation of the proteins by BPV E5.

In conclusion, RLR signalling pathway may be perturbed by BPV E5 oncoprotein, which allowed an adequate innate immune response to be not elicited against spontaneous bovine δPV infection, thus leading to persistent infection in the cells (**Figure 7**).

## Discussion

This study provides novel mechanistic insights into the potential role of E5 oncoprotein in dysregulating the host antiviral innate immune response in a spontaneous model of bovine papillomavirus disease. In urothelial cancer cells of cattle, mixed abortive and productive forms of BPV infections are known to occur (36). It is well known that physical status of the virus (episomal or integrated forms) may generate variability in the host innate antiviral response (15). Therefore, a protein can be both downregulated by episomal papillomavirus and upregulated by integrated papillomavirus in infected cells being its expression positively associated with the progression of lesions (37, 38). The lack of correlation between viral load and the expression of the studied antiviral immune response factors suggests that viral replication is not determinant in the E5-mediated downregulation of host immune responses. In fact, taking the example of the human papillomavirus, the viral genomes can persist episomally, without integration into host cell genomes in absence of functional E5 (39, 40). Therefore, our results are consistent with E5 functions beyond viral replication such as evasion of host immune responses and inhibition of provirus integration into host cell genome (41).

Our study showed, for the first time, that the E5 oncoprotein of bovine δPVs interacts with TRIM25, a key player in antiviral immunity (42), to hamper innate immune signalling pathway mediated by RIG-I and MDA5. These results are of interest as there are very limited, controversial *in vivo* studies based on the role of TRIM25 in RLR activation, which remains elusive (43, 44). However, the lack of direct evidence that bovine δPV E5-TRIM25 interaction could be responsible for the downregulation of downstream effectors of RLR signalling pathway, requires further experimental data by *in vitro* systems. E5 oncoprotein did not appear to influence the transcriptional activity of TRIM25; therefore, it is conceivable that E5 oncoprotein enhanced TRIM25 proteasomal degradation, which may hinder the activation of RIG-I and MDA5. It is well known that TRIM25 ubiquitinates and activates RLRs in a dose-dependent manner (45). Our results appear to be corroborated by experimental studies that showed that HPV oncoproteins could enhance the proteasomal degradation of TRIM25 (30). Furthermore, our study suggested the existence of multiple evasion mechanisms based on bovine δPV-mediated inhibition of key components of the RLR pathways. Indeed, E5-expressing cells showed a marked reduction in the transcriptional activity of both RIG-I and MDA5. Reduced RIG-I and MDA5 mRNA levels detected by real-time PCR suggested that some proteins of bovine δPVs could downregulate the transcriptional activity of RIG-I and MDA5, which allowed δPVs to impair the innate antiviral response, a prerequisite for persistent infection. Our results appeared to be strengthened by experimental data from *in vitro* studies in which HPV oncoproteins have been shown to act as transcriptional repressors of RIG-I and MDA5 to impair the viral host response during persistent infection (15, 46). RLRs catalyse the conversion of MAVS fibrils to prion-like aggregates. Although MAVS activation is a complex, multistep process, this conformational change of MAVS is essential for the recruitment of downstream signalling molecules (9). Not much is known about the mechanism(s) of how MAVS functions in antiviral signalling pathways (31); therefore, the activation mechanism of MAVS downstream pathways remains elusive (47). In our study, MAVS expression levels did not vary significantly. Many viruses block RLR-mediated immune signalling thus inhibiting host antiviral response without modifying MAVS expression levels (48). It is conceivable that in our spontaneous model of PV infection, the marked reduction in the expression levels of RIG-I and MDA5 may be responsible for the loss of conformational changes thus compromising the activation of MAVS, which is necessary for activating and propagating the antiviral signalling cascade. In addition, we found reduced expression levels of Sec13, which may contribute to further attenuation of MAVS downstream signalling. It has been suggested that Sec13 facilitates MAVS aggregation and ubiquitination and is thus required for RLR-MAVS-related antiviral responses (31). It has been shown that Sec13 expression correlates with MAVS activation. Indeed, the overexpression of Sec13 increases MAVS activation, whereas Sec13 downregulation attenuates MAVS activation (31). *In vitro* studies have shown that MAVS may serve as a scaffold to facilitate the interaction of TBK1 with IRF3 (33). MAVS has been shown to activate the transcription factor IRF3 through TBK1 (32). We found a marked reduction in the expression levels of total and phosphorylated TBK1, which may result in perturbation of IRF3 activation as TBK1 plays a crucial role in allowing efficient IRF3 phosphorylation in the IFN-producing pathways that require MAVS as the adaptor protein (32). Many viruses inhibit RIG-I/MAVS signalling by blocking TBK1 phosphorylation (49). It is conceivable that the E5 oncoprotein of bovine δPVs is a key player involved in the downregulation of TBK1 activation. Low expression of TBK1 has been shown to markedly reduce IFN1 induction (7) and proinflammatory macrophage (M1) polarisation (50). Furthermore, we found reduced expression levels of IRF3, which may hamper their interaction network, a critical step in the production of IFNs (33, 51). This study showed that canonical IKK complex composed of IKKα/IKKβ/IKKγ is downregulates at transcriptional level. IKK proteins are key components that are required for NF-kB activation, which allow us to suggest that BPV E5 oncoprotein could negatively influence NF-kB activation. Our findings corroborated experimental data showing that HPV oncoproteins are able to downregulate NF-kB activation thus contributing to virus escape from the immune system (52).

Bovine δPVs must escape innate immune surveillance to establish persistent infections and viral proteins may manipulate this process through several mechanisms. This study showed that similar to human PVs, bovine PVs perturb the RLR-mediated innate immune signalling pathway through the viral E oncoprotein, which is encoded in the early stages of PV infection. This perturbation results in an abnormal host antiviral response, which allows PVs to continue their infectious cycle leading to persistent viral infection. Bovine δPVs reduce the levels of the DNA sensors that can recognise BPVs, which can hamper pTBK1 signalling as well as the production of IFNs, similar to human PVs (53). IFN production plays a crucial role in the immune response against PV infection as IFNs promote the clearance of latent PV episomes in persistently infected cells (54) and/or rapid reduction in PV episome copies per cell (55). It has been suggested that basal cells in the initial infection usually contain low levels (around 100 copies per cell) of human and bovine PV episomes (56, 57). Animal cells that fail to resolve their infection and retain oncogene expression for years can facilitate tumourigenesis by BPVs (58).

In conclusion, bovine δPVs must escape innate immune surveillance to establish persistent infections, and viral proteins manipulate this process through several mechanisms. Despite the importance, molecular mechanisms for many bovine δPV oncoproteins remain poorly characterised, in part due to challenges in identifying their substrates. Therefore, further investigations aimed to clarify the functional role of viral oncoproteins at the intersection of immune evasion and aberrant proliferation of cells persistently infected by bovine δPVs, warrant future research.

## Data Availability Statement

The datasets presented in this study can be found in online repositories. The names of the repository/repositories and accession number(s) can be found in the article/**Supplementary Material**.

## Author Contributions

SR designed the experiments. FF, AC, VU, and PC carried out the experiments. SR, IG, and AC analysed data. SR wrote the manuscript. All authors contributed to the article and approved the submitted version.

## Funding

This research was partially supported by Regione Campania and Regione Basilicata. The funders of the work did not influence study design, data collection and analysis, decision to publish, or preparation of the manuscript.

## Conflict of Interest

The authors declare that the research was conducted in the absence of any commercial or financial relationships that could be construed as a potential conflict of interest.

## Supplementary Material

Supplementary Figure 1
**(A)** Real-time RT-PCR analysis of BPV-2 and BPV-13 E5 mRNA expression in healthy and infected bovine bladder samples. Lane MW: DNA molecular weight marker (100-base pair (bp) ladder); lanes 2 – 4: three representative infected bladder samples; lane 5: healthy bladder sample; lane C: no template control (no cDNA added). **(B)** The amplicon sequences showed 100% identity with BPV-2 E5 and BPV-13 E5 sequences deposited in GenBank (Accession numbers: M20219.1 and JQ798171.1, respectively). Electrophoretic representative data were obtained from three independent experiments.

Supplementary Figure 2
**(A)** TRIM25 cDNA amplification by PCR in normal and infected bovine urinary bladder samples compared with β-actin. Lane 1: molecular weight marker (DNA marker ladder); lanes 2-5: four representative infected bladder samples; lanes 6-9: healthy bladder samples; in the last channel: negative control (RNA without reverse transcriptase subjected to PCR analysis). **(B)** The lower part of the figure shows the alignment of the sequences, which revealed 100% identity with bovine TRIM25 transcript sequences deposited in GenBank (Bos taurus tripartite motif containing 25 (TRIM25), mRNA: NM_001100336.1).

Supplementary Figure 3
**(A)** RIG-I and MDA5 cDNA amplification by PCR in normal and infected bovine urinary bladder samples compared with β-actin. Lane 1: molecular weight marker (DNA marker ladder); lanes 2-5: four representative infected bladder samples; lanes 6-9: healthy bladder samples; in the last channel: negative control (RNA without reverse transcriptase subjected to PCR analysis). **(B)** The lower part of the figure shows the alignment of the sequences, which revealed 100% identity with bovine RIG-I and MDA5 transcript sequences deposited in GenBank (Bos taurus DExD/H-box helicase 58 (DDX58), transcript variant X1, mRNA: XM_002689480.6; Bos taurus interferon induced with helicase C domain 1 (IFH1), mRNA: XM_010802053.2).

Supplementary Figure 4 Relationship between viral load (x axis) and protein expression levels (y axis). A – Riplet; B -TRIM25; C – MDA5; D- RIG-I; E- Sec13; F- IRF3; G-TBK1; H-pTBK1. Pearsons’s p value was not statistically significant.

Supplementary Figure 5 Relationship between viral load (x axis) and mRNA expression levels (y axis). A – MDA5; B -RIG-I: C-TRIM25; D- IKKα; E- IKKβ; F- IKKγ; G – IFNβ. Pearsons’s p value was not of statistical significance.
