# Supplementary figures and images for: Bovine Delta Papillomavirus E5 Oncoprotein Interacts With TRIM25 and Hampers Antiviral Innate Immune Response Mediated by RIG-I-Like Receptors
Source: Front Immunol. 2021 Jun 10;12:658762. doi: 10.3389/fimmu.2021.658762 (PMC8223750; doi:10.3389/fimmu.2021.658762)

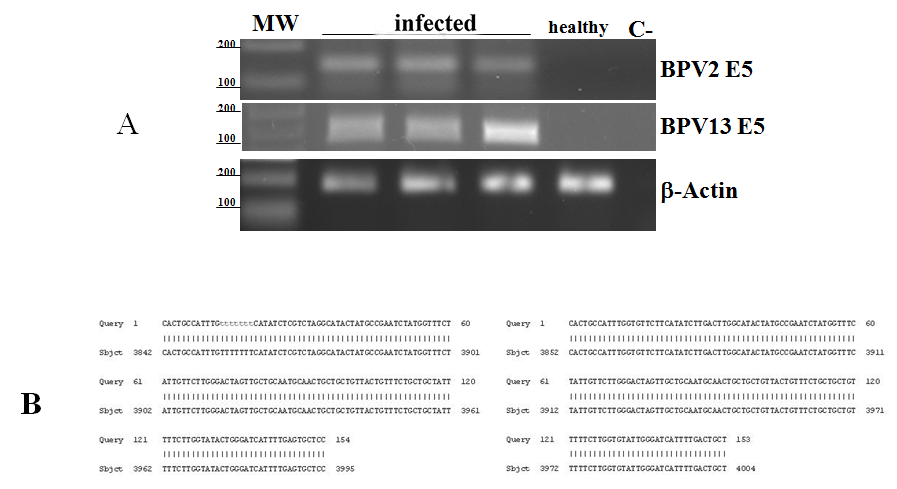

Supplement: Supplementary Figure 1 — (A) Real-time RT-PCR analysis of BPV-2 and BPV-13 E5 mRNA expression in healthy and infected bovine bladder samples. Lane MW: DNA molecular weight marker (100-base pair (bp) ladder); lanes 2 – 4: three representative infected bladder samples; lane 5: healthy bladder sample; lane C: no template control (no cDNA added). (B) The amplicon sequences showed 100% identity with BPV-2 E5 and BPV-13 E5 sequences deposited in GenBank (Accession numbers: M20219.1 and JQ798171.1, respectively). Electrophoretic representative data were obtained from three independent experiments. [file Image_1.tif]

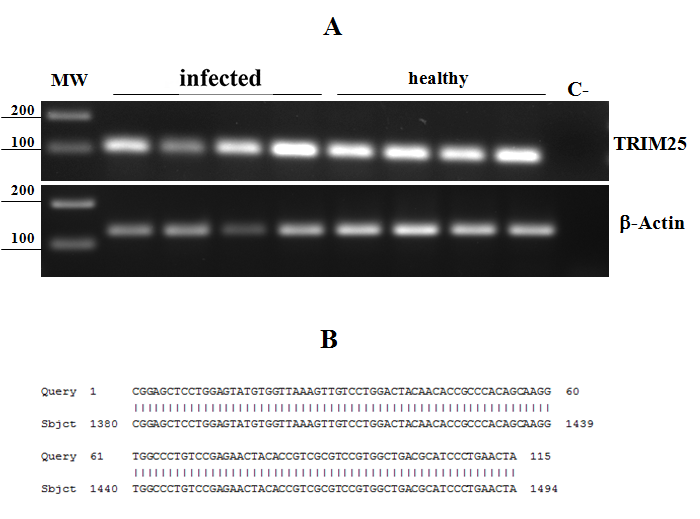

Supplement: Supplementary Figure 2 — (A) TRIM25 cDNA amplification by PCR in normal and infected bovine urinary bladder samples compared with β-actin. Lane 1: molecular weight marker (DNA marker ladder); lanes 2-5: four representative infected bladder samples; lanes 6-9: healthy bladder samples; in the last channel: negative control (RNA without reverse transcriptase subjected to PCR analysis). (B) The lower part of the figure shows the alignment of the sequences, which revealed 100% identity with bovine TRIM25 transcript sequences deposited in GenBank (Bos taurus tripartite motif containing 25 (TRIM25), mRNA: NM_001100336.1). [file Image_2.tif]

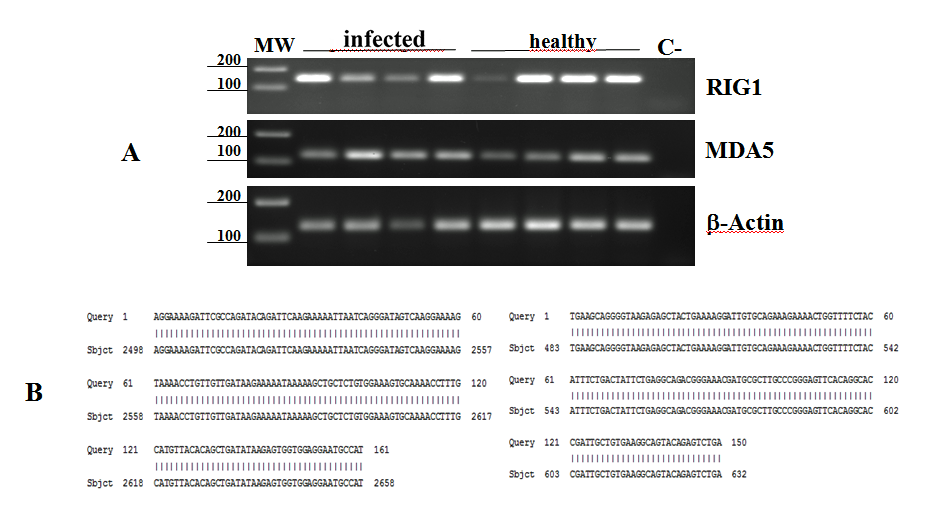

Supplement: Supplementary Figure 3 — (A) RIG-I and MDA5 cDNA amplification by PCR in normal and infected bovine urinary bladder samples compared with β-actin. Lane 1: molecular weight marker (DNA marker ladder); lanes 2-5: four representative infected bladder samples; lanes 6-9: healthy bladder samples; in the last channel: negative control (RNA without reverse transcriptase subjected to PCR analysis). (B) The lower part of the figure shows the alignment of the sequences, which revealed 100% identity with bovine RIG-I and MDA5 transcript sequences deposited in GenBank (Bos taurus DExD/H-box helicase 58 (DDX58), transcript variant X1, mRNA: XM_002689480.6; Bos taurus interferon induced with helicase C domain 1 (IFH1), mRNA: XM_010802053.2). [file Image_3.tif]
