# Supplementary material for: Bovine Delta Papillomavirus E5 Oncoprotein Interacts With TRIM25 and Hampers Antiviral Innate Immune Response Mediated by RIG-I-Like Receptors
Source: Front Immunol. 2021 Jun 10;12:658762. doi: 10.3389/fimmu.2021.658762 (PMC8223750; doi:10.3389/fimmu.2021.658762)
Supplement: Supplementary Figure 4 — Relationship between viral load (x axis) and protein expression levels (y axis). A – Riplet; B -TRIM25; C – MDA5; D- RIG-I; E- Sec13; F- IRF3; G-TBK1; H-pTBK1. Pearsons’s p value was not statistically significant. [file Image_4.pdf]

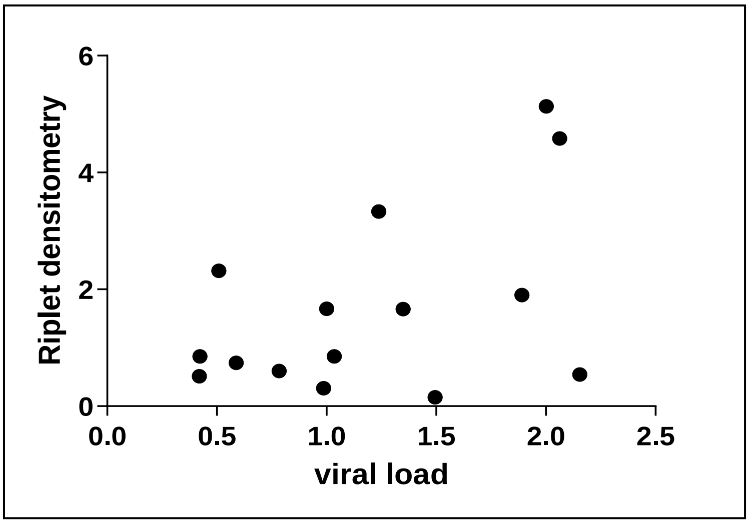

A

Pearson r	
r	0,4896
95% confidence interval	-0,03029 to 0,8010
R squared	0,2397
P value	
P (two-tailed)	0,064
P value summary	ns
Significant? (alpha = 0.05)	No
Number of XY Pairs	15

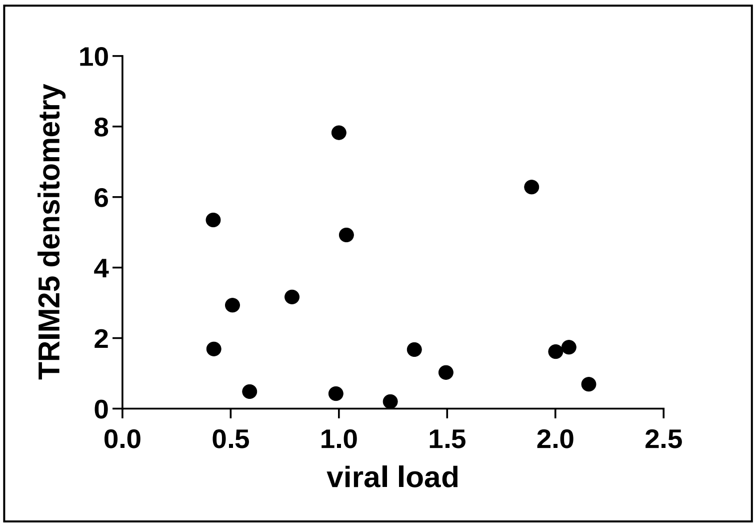

B

Pearson r	
r	-0,1514
95% confidence interval	-0,6159 to 0,3912
R squared	0,02292
P value	
P (two-tailed)	0,5902
P value summary	ns
Significant? (alpha = 0.05)	No
Number of XY Pairs	15

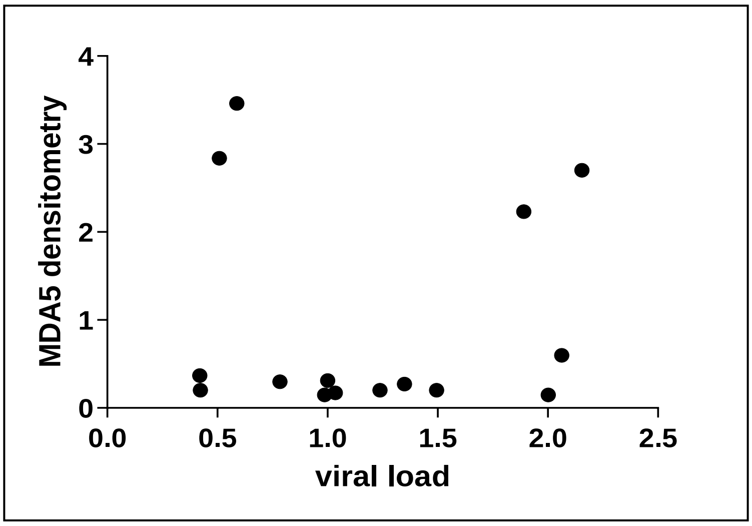

C

Pearson r	
r	0,01467
95% confidence interval	-0,5014 to 0,5230
R squared	0,0002153
P value	
P (two-tailed)	0,9586
P value summary	ns
Significant? (alpha = 0.05)	No
Number of XY Pairs	15

D

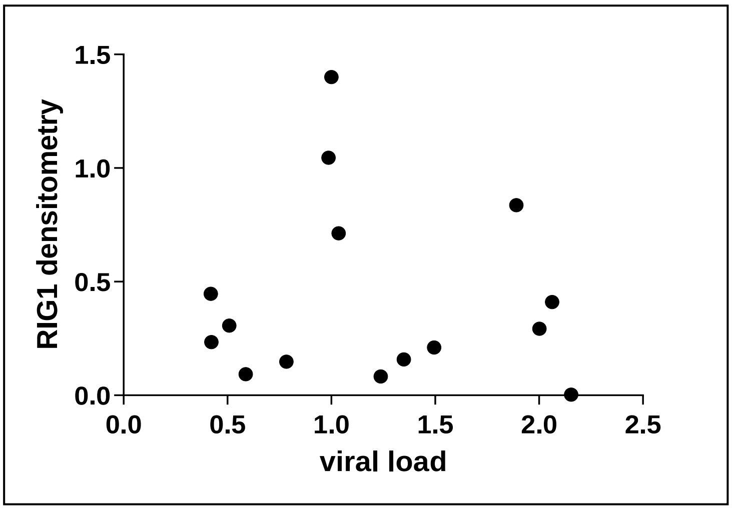

Pearson r	
r	-0,05707
95% confidence interval	-0,5532 to 0,4689
R squared	0,003257
P value	
P (two-tailed)	0,8399
P value summary	ns
Significant? (alpha = 0.05)	No
Number of XY Pairs	15

E

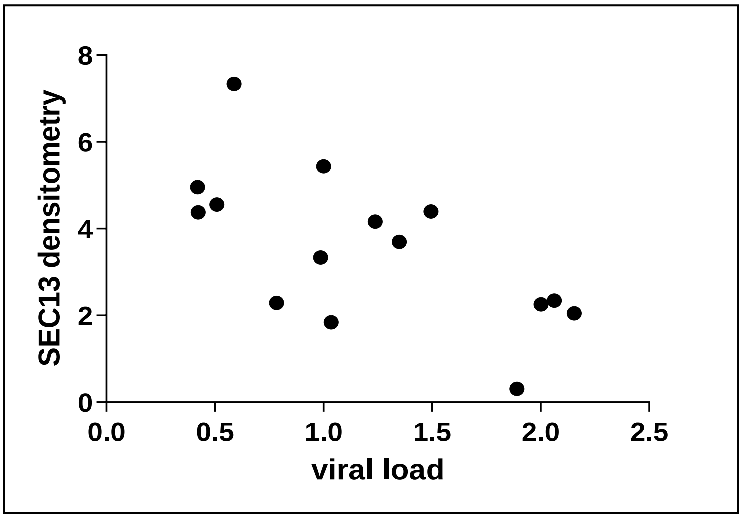

Pearson r	-0,06241
95% confidence interval	-0.5570 to 0.4648
P value (two-tailed)	0,8251
P value summary	ns
Is the correlation significant?	No
R squared	0,003896
Number of XY Pairs	15

F

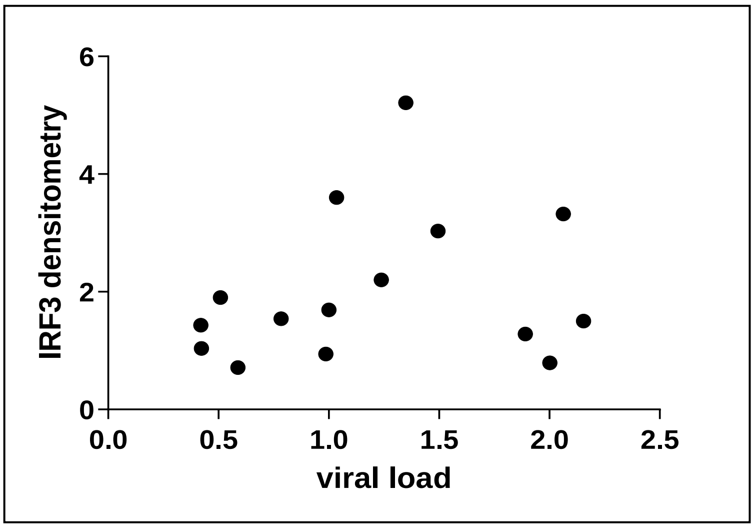

Pearson r	
r	0,2078
95% confidence interval	-0,3407 to 0,6508
R squared	0,04319
P value	
P (two-tailed)	0,4573
P value summary	ns
Significant? (alpha = 0.05)	No
Number of XY Pairs	15

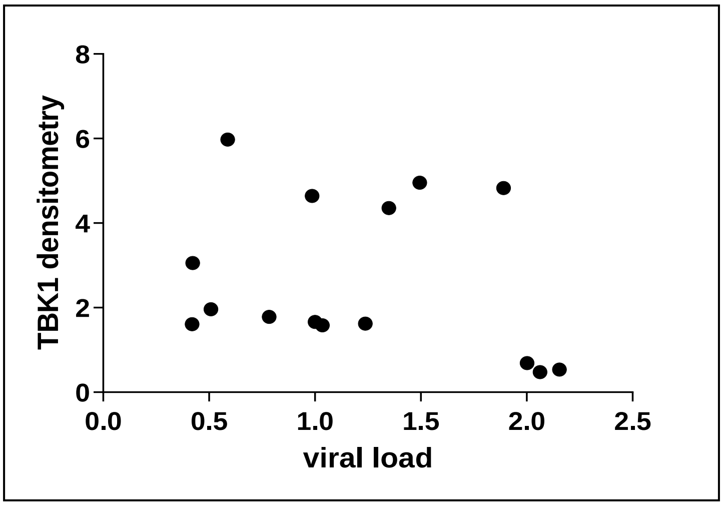

G

Pearson r	
r	-0,2442
95% confidence interval	-0,6724 to 0,3064
R squared	0,05963
P value	
P (two-tailed)	0,3804
P value summary	ns
Significant? (alpha = 0.05)	No
Number of XY Pairs	15

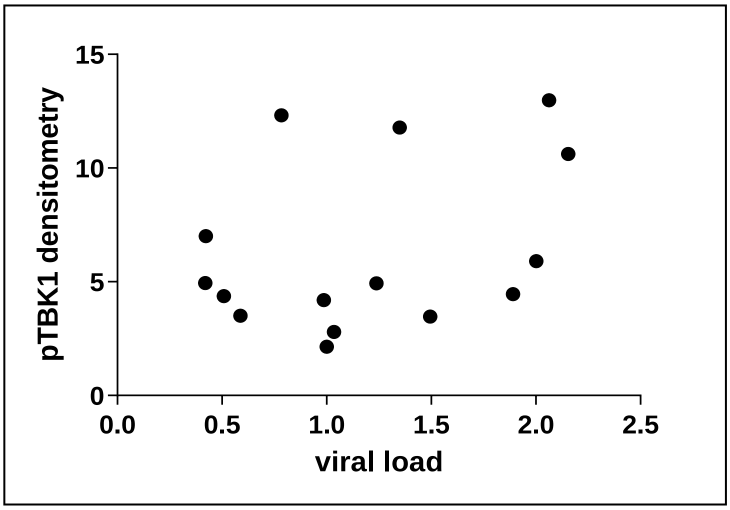

H

Pearson r	
r	0,3521
95% confidence interval	-0,1954 to 0,7323
R squared	0,124
P value	
P (two-tailed)	0,1981
P value summary	ns
Significant? (alpha = 0.05)	No
Number of XY Pairs	15
