# Supplementary material for: Bovine Delta Papillomavirus E5 Oncoprotein Interacts With TRIM25 and Hampers Antiviral Innate Immune Response Mediated by RIG-I-Like Receptors
Source: Front Immunol. 2021 Jun 10;12:658762. doi: 10.3389/fimmu.2021.658762 (PMC8223750; doi:10.3389/fimmu.2021.658762)
Supplement: Supplementary Figure 5 — Relationship between viral load (x axis) and mRNA expression levels (y axis). A – MDA5; B -RIG-I: C-TRIM25; D- IKKα; E- IKKβ; F- IKKγ; G – IFNβ. Pearsons’s p value was not of statistical significance. [file Image_5.pdf]

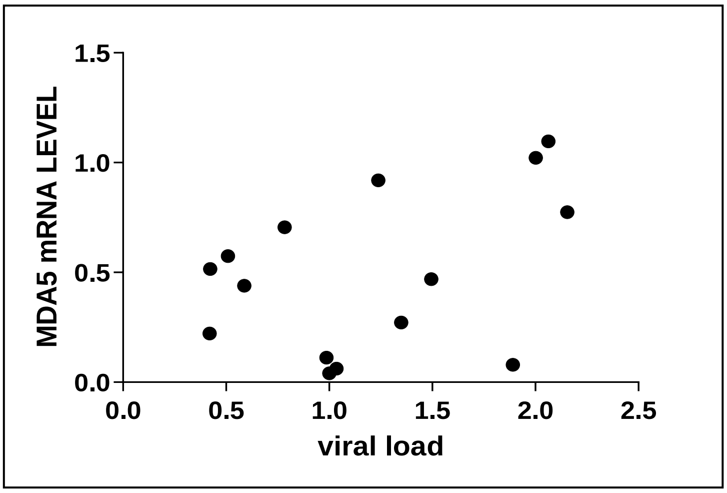


A

| | |
|---|---|
| Pearson r | |
| r | 0,4013 |
| 95% confidence interval | -0,1396 to 0,7578 |
| R squared | 0,1611 |
| | |
| P value | |
| P (two-tailed) | 0,1382 |
| P value summary | ns |
| Significant? (alpha = ( | No |
| | |
| Number of XY Pairs | 15 |

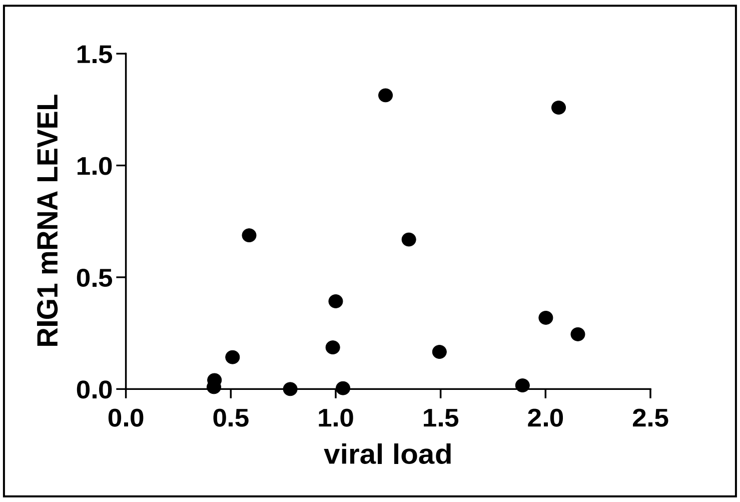


B

| | |
|---|---|
| Pearson r | |
| r | 0,3038 |
| 95% confidence interval | -0,2469 to 0,7061 |
| R squared | 0,09227 |
| | |
| P value | |
| P (two-tailed) | 0,2711 |
| P value summary | ns |
| Significant? (alpha = ( | No |
| | |
| Number of XY Pairs | 15 |

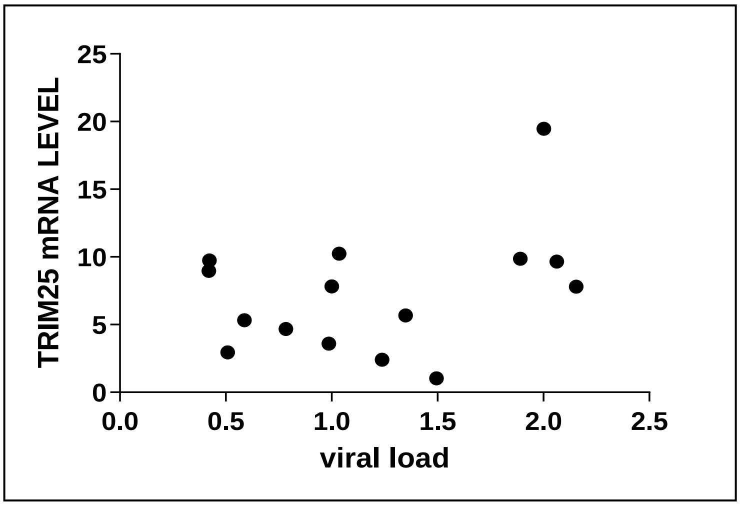


C

| | |
|---|---|
| Pearson r | |
| r | 0,3634 |
| 95% confidence interval | -0,1829 to 0,7383 |
| R squared | 0,1321 |
| | |
| P value | |
| P (two-tailed) | 0,183 |
| P value summary | ns |
| Significant? (alpha = ( | No |

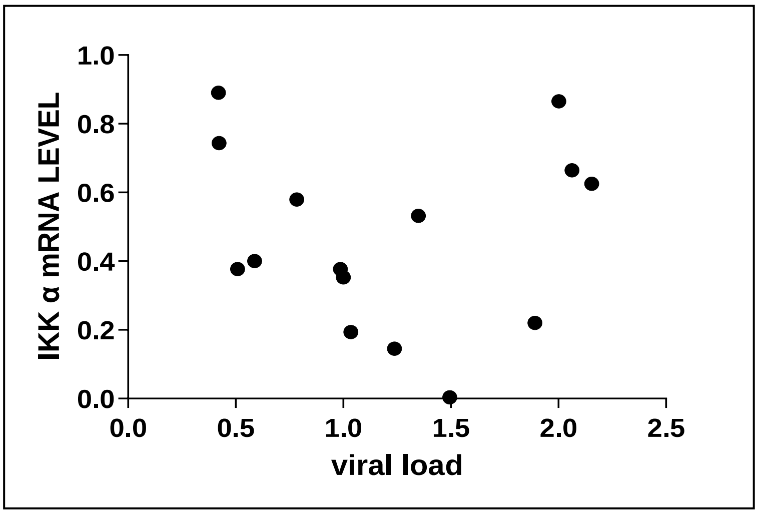


**D**

| | |
|---|---|
| Pearson r | |
| r | -0,03102 |
| 95% confidence interval | -0,5348 to 0,4890 |
| R squared | 0,000962 |
| | |
| P value | |
| P (two-tailed) | 0,9126 |
| P value summary | ns |
| Significant? (alpha = ( | No |
| | |
| Number of XY Pairs | 15 |

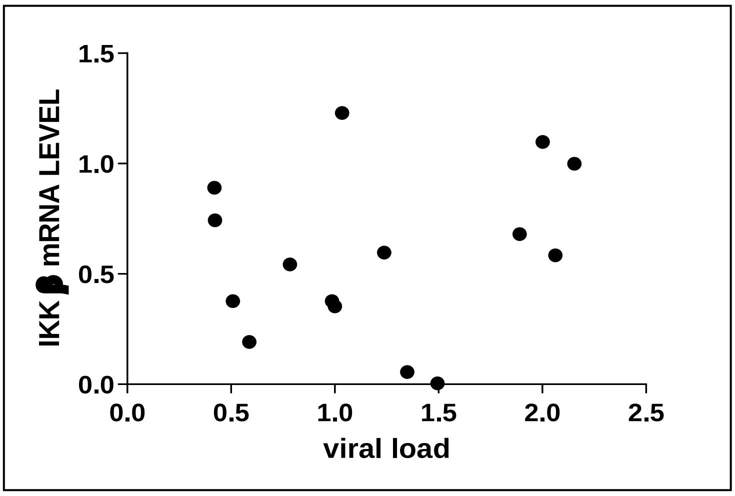


**E**

| | |
|---|---|
| Pearson r | |
| r | 0,2059 |
| 95% confidence interval | -0,3425 to 0,6496 |
| R squared | 0,0424 |
| | |
| P value | |
| P (two-tailed) | 0,4616 |
| P value summary | ns |
| Significant? (alpha = ( | No |
| | |
| Number of XY Pairs | 15 |

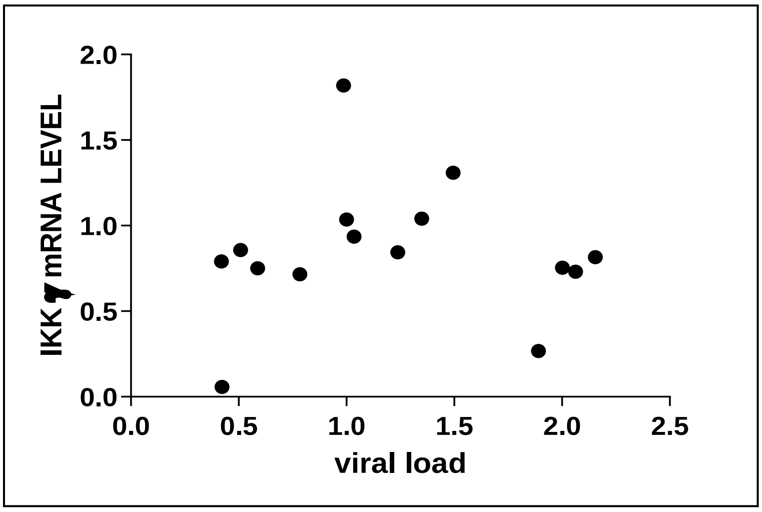


**F**

| | |
|---|---|
| Pearson r | |
| r | 0,2059 |
| 95% confidence interval | -0,3425 to 0,6496 |
| R squared | 0,0424 |
| | |
| P value | |
| P (two-tailed) | 0,4616 |
| P value summary | ns |
| Significant? (alpha = ( | No |
| | |
| Number of XY Pairs | 15 |

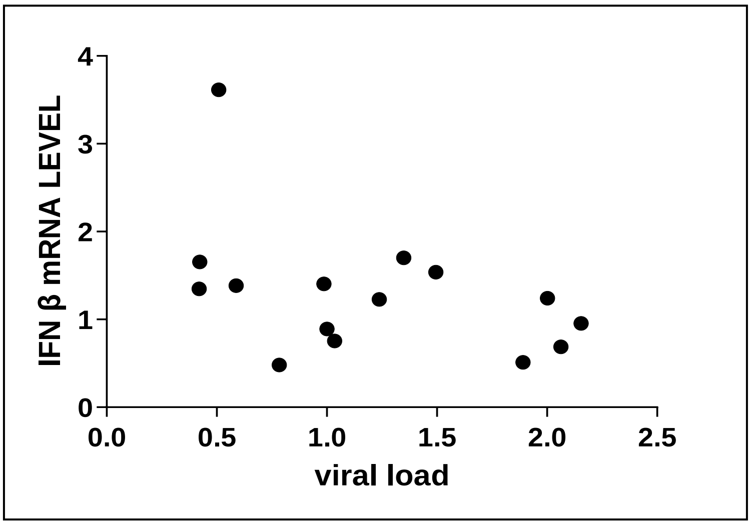


**G**

| | |
|---|---|
| Pearson r | |
| r | -0,4333 |
| 95% confidence interval | -0,7738 to 0,1015 |
| R squared | 0,1878 |
| | |
| P value | |
| P (two-tailed) | 0,1066 |
| P value summary | ns |
| Significant? (alpha = ( | No |
| | |
| Number of XY Pairs | 15 |
